# The expanding role of dopamine

**DOI:** 10.7554/eLife.15963

**Published:** 2016-04-21

**Authors:** Bradley B Doll, Nathaniel D Daw

**Affiliations:** 1 Center for Neural Science, New York University, New York, United States; 2 Department of Psychology and Princeton Neuroscience Institute, Princeton University, Princeton, United Statesndaw@princeton.edu

**Keywords:** dopamine, prediction error, single unit, Rat

## Abstract

Evidence increasingly suggests that dopaminergic neurons play a more sophisticated role in predicting rewards than previously thought.

**Related research article** Sadacca BF, Jones JL, Schoenbaum G. 2016. Midbrain dopamine neurons compute inferred and cached value prediction errors in a common framework. *eLife* **5**:e13665. doi: 10.7554/eLife.13665

**Image** Rats can learn to associate a clicker with a reward without experiencing the two at the same time

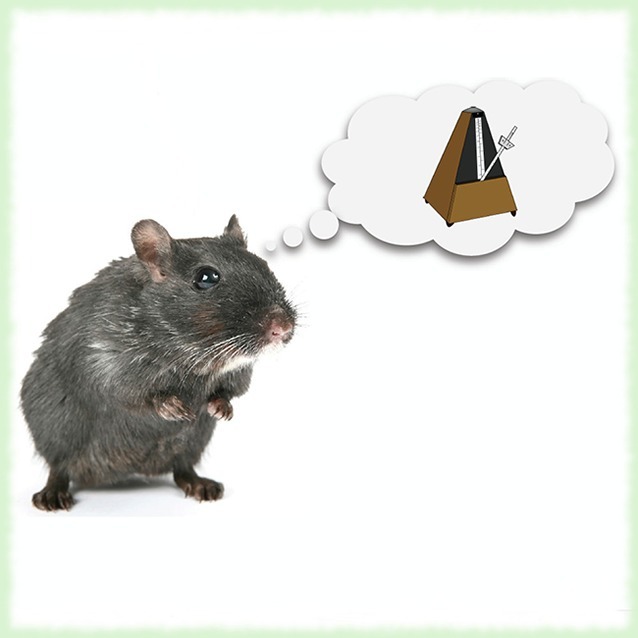

As any kindergarten instructor will tell you, reward is one of the most powerful teachers. Some of the earliest and most iconic examples of behavioral psychology concern how animals learn, from experience, which stimuli or actions accompany reward (Thorndike, 1898; Pavlov, 1927). A century later, computational neuroscientists have described neural circuits that underpin such learning. These are based on the mutual interactions between neurons that contain the neuromodulator dopamine and other neurons they connect with, particularly those in a brain region called the striatum. The dopaminergic neurons receive information about predicted rewards, and report back the mismatch between those expectations and the rewards actually received. These “reward prediction errors,” in turn, allow the predictions to be updated, a computation known as model-free learning.

The problem with this well-studied framework is that humans and rodents can learn about rewards in many ways other than by direct experience (Tolman, 1948). Computationally, these capabilities have been understood in terms of “model-based” learning methods, which draw on knowledge of task structure to anticipate possible rewards that have never been directly experienced. Due in part to the support for a tidy, closed-loop picture of dopamine’s involvement in reward prediction, researchers have tended to assume that such capabilities arise from some separate, more sophisticated brain system. Now, in eLife, Brian Sadacca, Joshua Jones and Geoffrey Schoenbaum indicate that these more sophisticated learning capabilities instead arise within – or at least impinge upon – the dopaminergic learning circuit itself (Sadacca et al., 2016).

Sadacca et al. – who are based at the National Institute on Drug Abuse, University of Maryland School of Medicine and Johns Hopkins School of Medicine – recorded spiking from dopaminergic neurons while rats performed a task designed to defeat simple model-free learning. The task, called sensory preconditioning, assesses the rats’ ability to associate a stimulus (for example, a clicker) with a reward without ever experiencing the two together (Figure 1). To do so, a rat needs to integrate experiences from two separate training phases. First, in a “pre-conditioning” phase, the clicker was paired with another neutral stimulus (for example, a tone); then, in a “conditioning” phase, the tone (but not the clicker) was paired with a reward. Not only was the clicker never paired with the reward, it was not even paired with a reward-predicting stimulus, since the tone’s relationship with reward was established at a later stage.

**Figure 1.**
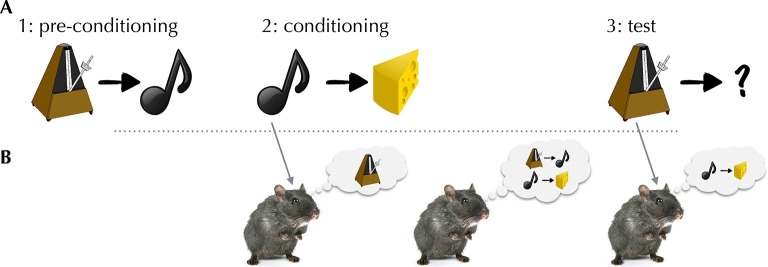
The activity of dopaminergic neurons helps rats to integrate separate experiences to predict when a reward will be given. (**A**) Schematic of the task used by Sadacca et al. In the pre-conditioning phase, rats learn to associate a clicker with a tone. In a subsequent conditioning phase, the rats learn to link the tone with a food reward. In the final test phase, the rats hear the clicker, and behave as if they expect a reward. (**B**) Three potential associative retrieval mechanisms that might support integrative inference about the stimulus. Left: during the conditioning phase, presenting the tone could call the clicker to mind, allowing both stimuli to be linked to a reward. Middle: after conditioning, the mental replay of experiences may permit the relationships between separate sets of stimuli to be learned. Right: in the test phase, the rats may make new inferences that cause the rats to expect a reward when they hear the clicker.

Nevertheless, in a test phase, rats demonstrated (by visiting a food cup where a reward had previously been delivered) that they associated the clicker with reward. This capability is well known; more surprising was that these reward predictions could also be seen in the responses of dopaminergic neurons to the clicker. This result complicates dual-system explanations, which state that model-based inference occurs separately from the dopaminergic reward learning system. It also speaks against the traditional closed-loop account of dopaminergic learning, in which the circuit should only know about reward predictions it has taught itself via direct pairings.

This result adds to a series of studies suggesting that responses in dopaminergic neurons and associated areas report more sophisticated reward predictions than theory suggests (Bromberg-Martin et al., 2010; Daw et al., 2011). Key challenges going forward are to understand how this information gets into the circuit, and what neural computations produce the information in the first place.

These questions might have the same answer if at least some of an animal’s sophisticated learning capacities actually build upon a dopaminergic foundation. This perspective is supported by work in humans that suggests that the dopamine system also helps to produce similar integrative inferences about rewards (Deserno et al., 2015; Sharp et al., 2015; Doll et al., 2016). There are several (not mutually exclusive) possibilities for how dopaminergic learning might contribute to these integrative predictions.

One possibility is that inferences made during the test phase cause the rats to expect a reward when they hear the clicker. Model-based learning theories envision that the brain retrieves successor stimuli (here, the clicker would evoke the tone) as a sort of mental simulation that helps to predict reward. Evidence of such retrieval has been demonstrated using fMRI in humans (Doll et al., 2015). Though this mechanism need not involve dopamine, it could: the usual dopaminergic learning circuit could map the evoked representations to a reward.

The association of the clicker with the reward could also have already been made earlier in the experiment. Though Sadacca et al. believe this is unlikely in their study, two broadly applicable mechanisms for this process have been suggested. One possibility, supported by a human fMRI study of a similar sensory preconditioning task (Wimmer and Shohamy, 2012), is that associations between the clicker and reward could already form in the conditioning phase (during which the rats learn to associate a tone with a reward). If presenting the tone called to mind the clicker, which preceded the tone in the first training phase, dopaminergic learning could associate the reward that followed with both stimuli.

Mentally rehearsing clicker-tone and tone-reward sequences after the conditioning phase (but before testing) could also allow the brain to learn the relationship between the clicker and reward. Replay of previously experienced events has been observed in neural recordings during sleep and quiet rest. If the brain treated such mock experiences like real ones – allowing them to drive dopaminergic responding and learning – this too could drive the integrative association (Gershman et al., 2014).

In all, the venerable framework of dopaminergic reward learning may have more explanatory power than originally thought, as the results of Sadacca et al. suggest that it might point toward explanations even for cases that were thought to challenge it.
